# Abatacept and the risk of malignancy: a meta-analysis across disease indications

**DOI:** 10.1093/rheumatology/keaf114

**Published:** 2025-02-24

**Authors:** Benjamin P Zuckerman, Mark Gibson, Ritika Roy, Mark Hughes, Daksh Mehta, Zijing Yang, Maryam Adas, Kenrick Ng, Mark D Russell, Andrew Cope, Sam Norton, James Galloway

**Affiliations:** Centre for Rheumatic Diseases, King’s College London, London, UK; Centre for Rheumatic Diseases, King’s College London, London, UK; Guy’s King’s and St Thomas’ Medical School, King’s College London, London, UK; Guy’s King’s and St Thomas’ Medical School, King’s College London, London, UK; Guy’s King’s and St Thomas’ Medical School, King’s College London, London, UK; Centre for Rheumatic Diseases, King’s College London, London, UK; Centre for Rheumatic Diseases, King’s College London, London, UK; Department of Medical Oncology, University College London, London, UK; Centre for Rheumatic Diseases, King’s College London, London, UK; Centre for Rheumatic Diseases, King’s College London, London, UK; Guy’s King’s and St Thomas’ Medical School, King’s College London, London, UK; Guy’s King’s and St Thomas’ Medical School, King’s College London, London, UK; Centre for Rheumatic Diseases, King’s College London, London, UK; Guy’s King’s and St Thomas’ Medical School, King’s College London, London, UK

**Keywords:** rheumatoid arthritis, psoriatic arthritis, biological therapies, abatacept, malignancy

## Abstract

**Objectives:**

To estimate the association between abatacept use and the incidence of malignancy excluding non-melanomatous skin cancers (NMSCs).

**Methods:**

Systematic database searches were performed, to April 2024, to identify phase II/III/IV randomized clinical trials (RCTs), long-term extension (LTE) and observational cohort studies of abatacept in people with rheumatoid arthritis and psoriatic arthritis. Network and pairwise meta-analyses were performed to estimate incidence rate ratios (IRRs) for malignancy excluding NMSC, comparing abatacept with placebo and tumour necrosis factor inhibitors (TNFi) in RCT/LTE studies. Pairwise meta-analyses evaluated the same outcome in observational studies, comparing abatacept with conventional synthetic DMARDs (csDMARDs) and biologic/targeted synthetic disease modifying antirheumatic drugs (b/tsDMARDs).

**Results:**

In 18 eligible RCTs and 10 LTE studies, there were 15 535 person-years of exposure to abatacept, 1495 to placebo and 733 to TNFi. In network meta-analyses of combined RCT/LTE data, the incidence of all malignancies excluding NMSCs was not significantly different between abatacept and placebo (IRR 0.58; 95% CI 0.32–1.09) or TNFi (IRR 0.72; 95% 0.27–1.87). In observational data, the incidence of malignancy was higher with abatacept, relative to other b/tsDMARDs (IRR 1.21; 95% CI 1.15–1.28), but not significantly different compared with csDMARDs (IRR 0.97; 95% CI 0.90–1.06).

**Conclusions:**

Abatacept was associated with a higher incidence of malignancy compared with other b/tsDMARDs in observational studies, but not when compared with placebo or TNFi in RCT/LTE data. Further pharmacovigilance data is essential to help elucidate whether abatacept modifies cancer risk.

**PROSPERO registration number:**

CRD42023382314.

Rheumatology key messages

- Abatacept was not linked to higher malignancy risk in clinical trial data.
- Observational studies found increased malignancy risk with abatacept *vs* other b/tsDMARDs, but not csDMARDs.

## Introduction

Abatacept, a selective co-stimulation modulator of T cells binds to CD80/86 ligands on antigen-presenting cells, inhibiting their engagement with cytotoxic T-lymphocyte-associated antigen 4 (CTLA-4) on T cells; a process integral to T-cell activation [1]. This mechanism is pivotal in conditions such as rheumatoid arthritis (RA), where abatacept has demonstrated notable efficacy [2–6]. Licensed treatment indications for abatacept have since expanded to include psoriatic arthritis (PsA) and juvenile idiopathic arthritis (JIA). More recently, abatacept has been studied in individuals with RA, with both the APIPPRA and ARIAA trials meeting their primary endpoints, suggesting a potential role for co-stimulation modulation in delaying the onset of RA in at-risk individuals [7, 8].

Inhibition of CTLA-4 has been utilized to promote anti-tumour immunity: ipilimumab, a fully human monoclonal antibody (IgG1) that inhibits CTLA-4, is widely used in the treatment of malignant melanoma and prostate cancer [9]. It is therefore biologically plausible that limiting co-stimulation of T cells may reduce anti-tumour immunity and increase malignancy risk. Patients with RA have an increased risk of non-melanomatous skin [10], solid organ cancers [11] and lymphoma [11] compared with the general population. This is thought to be due to a combination of shared environmental risk factors (e.g. cigarette smoking), chronic inflammation and the immunosuppressive effects of treatments [12–15].

The long-term safety profile of abatacept, particularly concerning malignancy risk, remains unclear. Several studies have shown a favourable long-term safety profile, finding no significantly increased risk of malignancy with abatacept [16–19] while other studies have reported an increased risk of malignancy associated with the use of abatacept [20–22]. In a retrospective observational study of US healthcare databases, a small but significantly increased risk of malignancy was found to be associated with abatacept use when compared with other biologic or targeted synthetic disease-modifying anti-rheumatic drugs (b/tsDMARDs) [hazard ratio (HR) 1.09, 95% confidence interval (CI) 1.02–1.16] [22]. Similarly, in another US-based cohort study, when used as the first b/tsDMARD in the treatment of RA, abatacept was associated with a significantly increased risk of all cancers (HR 1.17, 95% CI 1.06–1.30) and non-melanoma skin cancer (NMSC) when compared with other b/tsDMARDs (HR 1.20; 95% CI 1.03–1.39) [20]. Further to this, an evaluation of data from the Bristol Myers Squibb abatacept development programme suggested that abatacept significantly increased the risk of NMSC compared with conventional synthetic DMARDs (csDMARDs) [pooled rate ratios (RR) 1.84, 95% CI 1.00–3.37], but not b/tsDMARDs (RR 1.11, 95% CI 0.98–1.26) [21]. In this study, the risk of NMSC associated with abatacept administration was driven by data from observational studies, rather than RCT data [21].

In the presence of conflicting evidence, our objective was to meta-analyse all available data, to evaluate whether abatacept is associated with an increased risk of malignancy, compared with placebo, csDMARDs and other b/tsDMARDs.

## Methods

### Database search strategy

A systematic literature search was conducted using MEDLINE, Embase and Cochrane databases to identify RCTs, long-term extension (LTE) and observational cohort studies of abatacept in people with RA or PsA. Search terms are provided within the Supplementary Appendix, available at *Rheumatology* online. The search was limited to articles published from database inception to 30 April 2024. Additional trials were searched for in study references and trials databases. The search was performed in accordance with the Preferred Reporting System for Systematic Reviews (PRISMA) and registered with the International Prospective Register of Systematic Reviews (PROSPERO registration ID: CRD42023382314) [23].

### Eligibility criteria and study selection

Studies were eligible if they reported Phase II/III/IV RCTs, LTE, prospective or retrospective observational cohort studies of abatacept in adults with RA or PsA (Table 1). Studies performed in individuals with pre-RA diagnoses were also eligible for inclusion in sensitivity analyses. We did not include studies of abatacept in JIA due to the much lower baseline malignancy rate in this population [38]. Eligible comparators were placebo, methotrexate (MTX) monotherapy and TNFi for RCT and LTE studies. For observational studies, eligible comparators were grouped into conventional synthetic DMARDs (csDMARDs) and b/tsDMARDs, to align with how data were presented in these studies; a list of the other b/tsDMARDs and csDMARDs used in the observational studies can be found in the Supplementary Material, available at *Rheumatology* online. For LTE studies where only interim data were published, or where the same dataset was reported on more than once, the most recently published data were included. Studies not reporting data on malignancy outcomes were not eligible. Case report, case-control and cross-sectional studies were excluded.

**Table 1.** RCT/LTE characteristics

Author (study year/name/reference)	Study location	Study phase	Study duration, weeks	Disease	Doses included in analyses	Comparators	Number of participants	Mean age, years	Female participants, %	Reference
Mease (2011) (NCT00534313)	Worldwide	2b	24	PsA	10/30 mg/kg	PBO	83	53	45	[24]
Strand (2020) (ASTRAEA)	Worldwide	LTE	52	PsA	125 mg	PBO	474	51	57	[25]
Schiff (2009) (ATTEST)	Worldwide	LTE	80	RA	10 mg/kg	PBO, IFX	528	49	83	[26]
Westhovens (2009) (NCT00162266)	Worldwide	LTE	364	RA	10 mg/kg	PBO	270	56	75	[27]
Conaghan (2010) (ASSET)	Europe	3b	16	RA	10 mg/kg	PBO	27	52	59	[28]
Emery (2014) (AVERT)	Worldwide	3b	52	RA	125 mg	MTX	119	45	77	[4]
Emery (2023) (AVERT-2)	Worldwide	3b	182	RA	125 mg	PBO	752	49	77	[29]
Weinblatt (2012) (AMPLE)	N./S. America	3	104	RA	125 mg	ADA	318	51	81	[30]
Westhovens (2008) (AGREE)	Worldwide	LTE	104	RA	10 mg/kg	PBO	665	50	77	[27]
Kremer (2009) (AIM)	N. America	LTE	204	RA	10 mg/kg	PBO	1165	52	22	[31]
Weinblatt (2009) (ASSURE)	N. America	LTE	259	RA	10 mg/kg	PBO	1579	52	82	[32]
Blanco (2015) (NUURTURE 1)	Worldwide	3	52	RA	10 mg/kg	PBO	138	52	78	[33]
Rigby (2019) (NCT02557100)	N./C. America	LTE	48	RA	125 mg	ADA	112	47	73	[34]
Hetland (2018) (NORDSTAR)	Europe	4	24	RA	125 mg	CERT	204	55	69	[35]
Takeuchi (2007) (NCT00345748)	Japan	2	24	RA	10 mg/kg	PBO	62	53	80	[36]
Emery (2008) (ADJUST)	Worldwide	2	24	RA	10 mg/kg	PBO	28	44	71	[3]
Genovese (2009) (ATTAIN)	N. America	LTE	284	RA	10 mg/kg	PBO	685	53	77	[5]
Westhovens (2012) (NCT00254293)	N. America, Europe	LTE	311	RA	10 mg/kg	PBO	130	56	75	[37]
Cope (2024) (EduraCT[2013–003413–18])	Europe	2b	104	pre-RA	125 mg	PBO	110	48	76	[8]
Rech (2024) (EudraCT[2014–000555–93])	Europe	2	78	pre-RA	125 mg	PBO	49	51	63	[7]

For study location, worldwide refers to a study that was performed in three or more continents. Mean age and the proportion of female participants are shown for pooled abatacept groups of each study, where available. Study duration refers to the maximum duration of follow-up included in meta-analyses. Number of participants refers to the total participant numbers included in these analyses from all relevant study arms; for LTE studies not reporting comparator data, then comparator data from the original RCTs was incorporated.

LTE: long-term extension study; PsA: psoriatic arthritis; PBO: placebo; RA: rheumatoid arthritis; RCT: randomized controlled trial.

Records were managed in Rayyan (Cambridge, MA, USA). Study titles and abstracts were screened independently by three investigators (B.P.Z., M.G., R.R.) and the full texts of relevant studies were retrieved and assessed for eligibility. Disagreements were resolved through consensus discussion, with involvement of additional reviewers if required (M.D.R., J.G.).

### Data collection

Data were identified from publications, with cross-referencing to published information in clinicaltrials.gov. Data extracted included: study characteristics; participant demographics; intervention and comparator arms; person-years of exposure; and malignancy events (benign tumours, when specified, were not included). For observational cohort studies, data on concomitant csDMARD usage were extracted. In studies in which person-years of exposure were not reported, we calculated exposure from per-protocol participant disposition. Per-protocol disposition was selected over intention-to-treat disposition, as our primary objective was to assess medication safety.

Risk of bias was assessed using the Cochrane Risk of Bias-2 (ROB2) tool for RCTs [39] and Newcastle-Ottawa scale for observational cohort studies [40]. This was performed independently by investigators for each eligible RCT/LTE and observational cohort study (B.P.Z., M.G., M.H., D.M.), with disagreements resolved by involvement of a third reviewer (M.D.R.).

### Outcomes

The primary outcome was the incidence of all malignancies excluding NMSC. The risk of NMSC was recently meta-analysed, and therefore not included in these analyses [21].

### Statistical analysis

Meta-analyses were performed to estimate the risk of malignancy between abatacept and comparator arms. Network meta-analysis was employed, utilizing restricted maximum likelihood models, to assess outcomes by integrating both direct and indirect comparisons between treatments. The between-treatment IRRs for malignancies, along with their 95% confidence intervals, were visually represented using forest plots. Network plots were used to display the number of studies for each treatment and comparison. To address studies with zero events in one or more groups, a fixed continuity corrections of 0.1 was applied to each arm. The ranking of each drug was determined by estimated probabilities derived from the network meta-analysis parameters, and these rankings were synthesized by calculating the surface under the cumulative ranking curve (SUCRA). Network meta-analysis consistency assumptions were tested at the overall level (Wald test for inconsistency) and for each treatment comparison (node-splitting model).

Pairwise meta-analysis was conducted separately for RCT data alone, RCT and LTE data combined, and observational data alone. Pooled, weighted incidence rates and incidence rate ratios (IRR) for malignancies were reported. In LTE studies not reporting comparator data, comparator data from the original RCTs was included for comparison. IRRs were calculated using the random-effects DerSimonian and Laird method and compared graphically using forest plots. Heterogeneity was reported using I^2^ statistics. A fixed continuity correction of 0.1 was used for study arms with zero events, whereby group-specific adjustment values were estimated to account for imbalance between treatment and comparator groups sizes in studies [41]. A treatment arm continuity correction for studies with zero events was used in sensitivity analyses.

Funnel plots and Egger’s test for funnel asymmetry were performed to assess publication bias in our primary meta-analysis. Sensitivity analyses included: (i) analysis of studies conducted in participants with RA only; (ii) analysis of studies including pre-RA populations; (iii) ‘leave-one-out’ meta-analysis to examine the robustness of the overall results by systematically removing one study at a time and recalculating the overall effect size to identify any study that disproportionately influenced the outcomes; (iv) basic outlier removal and influence analyses to investigate the effect of influential studies on pooled estimates; (v) adding extra events to the exposure arms of the five trials with the greatest weight; and (vi) exclusion of studies assessed to be at high risk of bias.

Statistical analyses were conducted using dmetar, meta and metafor packages in R (R version 4.2.1., Vienna R Foundation for Statistical Computing; 2019) [42].

### Data availability and ethics

The data utilized in this study are freely available online; no ethnical approval was required as per UK Health Research Authority guidance.

## Results

### Randomized controlled trials and long-term extension studies

Eighteen RCTs were included, ten of which had LTE studies. Details of included studies and a flowchart of study selection is shown in Table 1 and Supplementary Fig. S1, available at *Rheumatology* online. Of the 18 eligible RCTs, 15 had placebo arms and four had TNFi groups (adalimumab *n* = 2; infliximab *n* = 1; certolizumab pegol *n* = 1). From pooled RCT and LTE data, there were 15 535 person-years of exposure to abatacept groups (mean: 885 person-years per study; *n* = 7822 participants; mean follow-up 126 weeks ± 117); 1495 person-years of exposure to placebo groups (mean: 83 person-years per study; *n* = 2283 participants; mean follow-up 49 weeks ± 44); and 733 person-years of exposure to TNFi groups (mean: 41 person-years per study; *n* = 737 participants; mean follow-up 19 weeks ± 48).

Of the 18 included RCTs, 11 (61.0%) were considered to have some concern of bias and five (28.0%) had at least one domain with a high risk of bias (Supplementary Table S1, available at *Rheumatology* online).

Across all 18 eligible RCTs, there were 26 malignancy events, corresponding to an incidence rate (IR) of 1.67 cancers per 100 person-years of exposure. There were 247 malignancy events across combined RCT and LTE data (IR of 1.36 cancers per 100 person-years).

### Observational studies

Eleven observational cohorts from six published studies were included, with data from registries (*n* = 5), claims databases (*n* = 4) and single centre cohorts (*n* = 2) (Supplementary Tables S2–S4, available at *Rheumatology* online). A flowchart of study selection is shown in Supplementary Fig. S2, available at *Rheumatology* online. Comparators included: other b/tsDMARDs (*n* = 2), csDMARDs (*n* = 1) or both (*n* = 3). There were 104 809 person-years of exposure to abatacept groups (mean: 9528 person-years per study; *n* = 53 260 participants; mean follow-up 123 weeks; standard deviation for follow-up 96 weeks); 717 528 person-years of exposure to other b/tsDMARDs (mean: 71 753 person-years per study; *n* = 195 109 participants; mean follow-up 173 weeks; standard deviation for follow-up 58 weeks); and 1 460 223 person-years of exposure to csDMARDs (mean: 208 603 person-years per study; *n* = 312 578 participants; mean follow-up 156 weeks; standard deviation for follow-up 95 weeks). There were 28 030 malignancies across all observational studies (IR of 1.70 cancers per 100 person-years). From the included observational studies, the six included studies demonstrated a low risk of bias (Supplementary Table S5, available at *Rheumatology* online).

### Network meta-analysis

**Figure 1.**
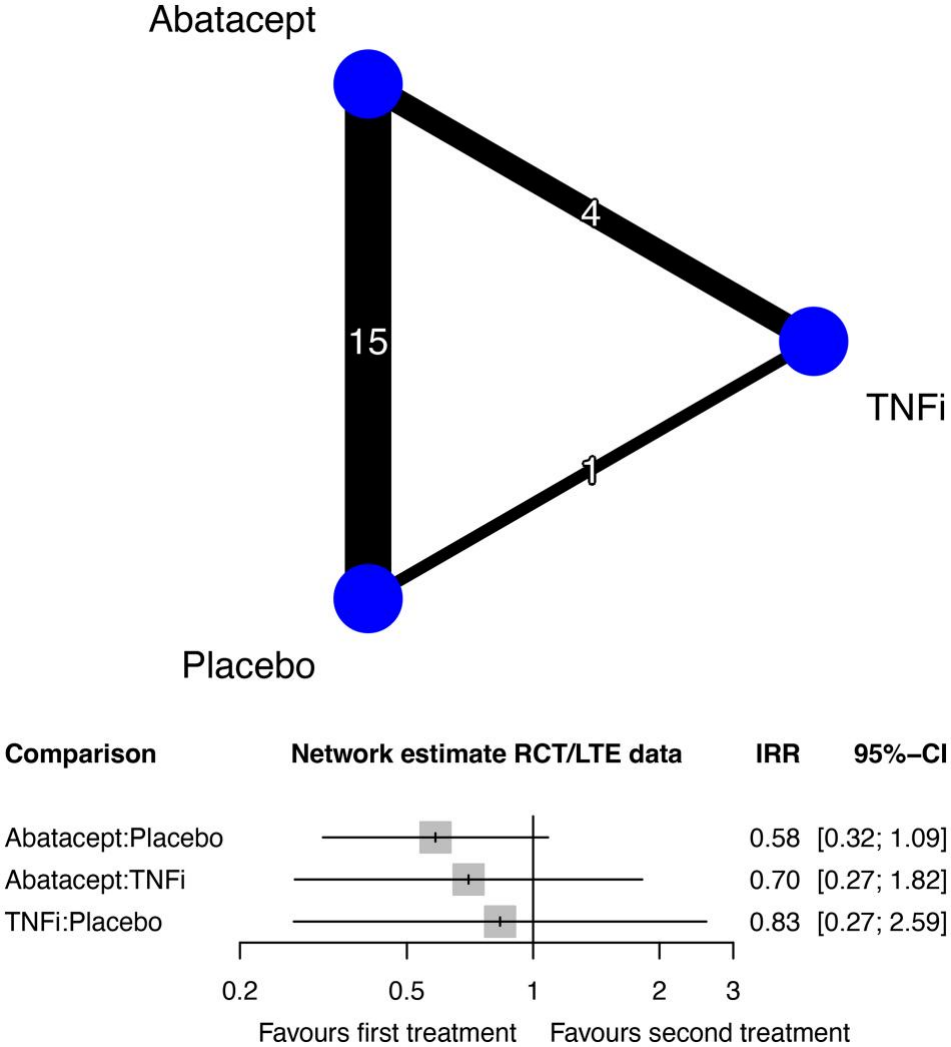
Network plot, depicting the number of studies for each treatment (node size) and number of treatment comparisons (edge thickness) in eligible RCT and LTE studies. In LTE studies without long-term comparator data, comparator data from the original RCTs were included. Network meta-analysis estimates of the risk of all malignancies excluding non-melanomatous skin cancers between study treatments expressed as incidence rate ratios with 95% CIs and depicted graphically as a forest plot. CI: confidence interval; LTE: long-term extension; RCT: randomized clinical trial; TNFi: tumour necrosis factor-a inhibitor

Malignancy risk was ranked across all treatments using SUCRA: abatacept was associated with the lowest risk of malignancy, followed by TNFi and placebo in the network meta-analysis of RCT and combined RCT/LTE data (Supplementary Tables S6 and S7, available at *Rheumatology* online). Treatment comparisons are shown in network plots (Fig. 1). Inconsistency was absent in the traits of network meta-analysis assumptions.

### Malignancy risk comparing abatacept with placebo

Malignancy risk estimates determined by network meta-analysis of all eligible RCT/LTE data are presented in Fig. 1. There was no significant difference in malignancy risk between abatacept and placebo (IRR 0.58; 95% CI 0.32–1.09) (Fig. 1). The pairwise meta-analysis corroborated this result (IRR 0.58; 95% CI 0.31–1.08) (Supplementary Fig. S3, available at *Rheumatology* online). In analyses of RCT data alone, there were no significant differences in the risk of malignancies between abatacept and placebo, into both network meta-analysis (IRR 0.54; 95% confidence CI 0.09–3.09) (Supplementary Fig. S4, available at *Rheumatology* online) and pairwise meta-analysis (IRR 0.40; 95% CI 0.06–2.27) (Supplementary Fig. S3, available at *Rheumatology* online).

### Malignancy risk comparing abatacept with TNFi

There were no significant differences in malignancy risk between abatacept and TNFi in network meta-analyses (IRR 0.70; 95% 0.27–1.81) (Fig. 1). Pairwise meta-analysis of combined RCT/LTE data (IRR 0.72; 95% 0.27–1.87) (Supplementary Fig. S5, available at *Rheumatology* online) and eligible RCT data only demonstrated comparable results (IRR 0.96; CI 0.31–2.99) (Supplementary Fig. S5, available at *Rheumatology* online).

### Observational studies

**Figure 2.**
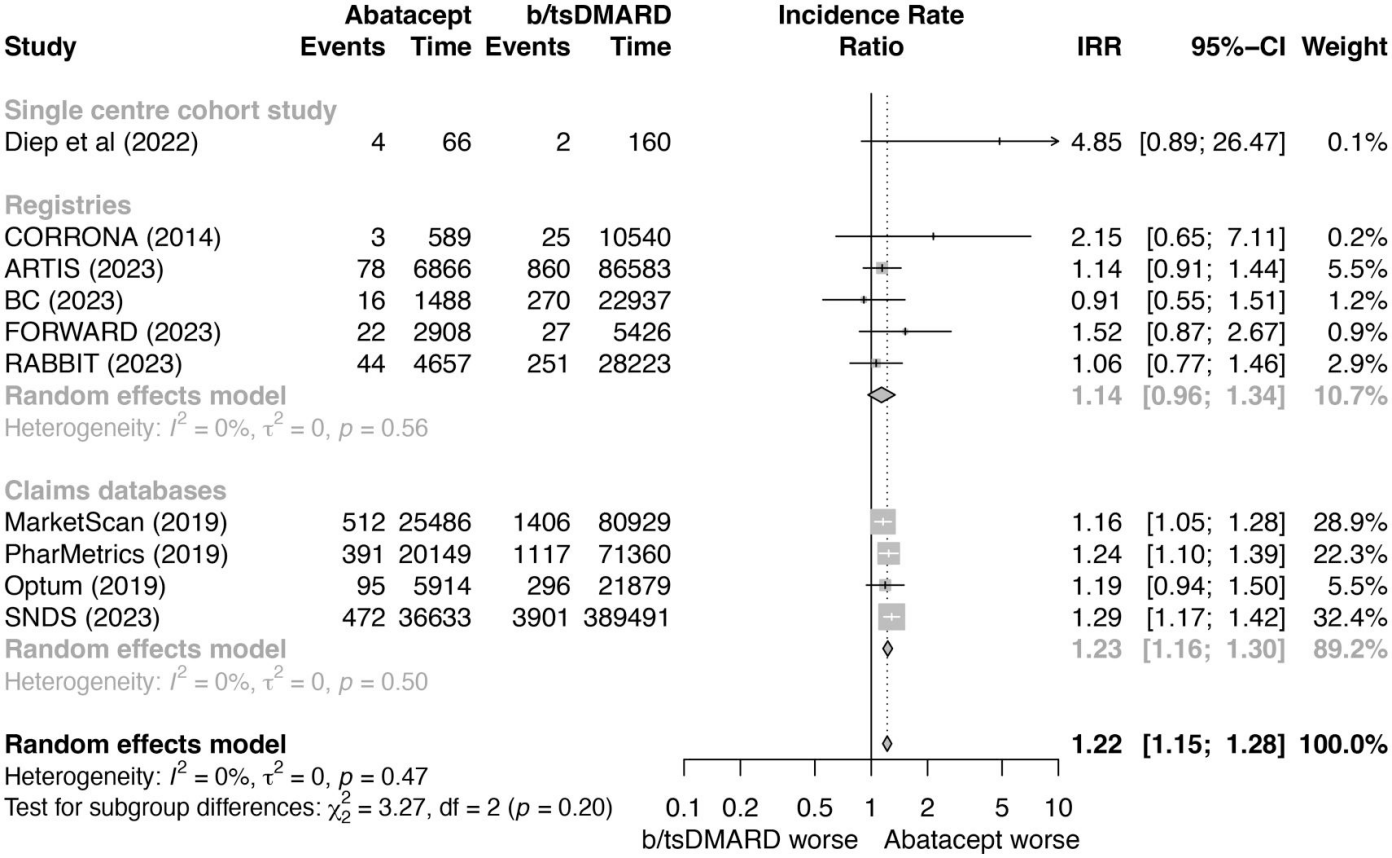
Pairwise meta-analysis of the risk of all malignancies excluding non-melanomatous skin cancers between abatacept and biologic/targeted synthetic disease modifying anti-rheumatic drug groups of eligible observational cohort studies; expressed as incidence rate ratios with 95% CIs and depicted graphically as a forest plot. Exposure is reported in person-years. The relative weighting of each study from a random-effects model is shown. A fixed continuity correction of 0.1 was applied to all studies with zero events. Heterogeneity between studies was assessed using I2 statistics. b/tsDMARD, biologic/targeted synthetic disease modifying anti-rheumatic drugs; CI, confidence interval

Pairwise meta-analysis of observational studies showed a significantly greater incidence of all malignancies excluding NMSC with abatacept compared with pooled other b/tsDMARDs (IRR 1.21; 95% CI 1.15–1.28) (Fig. 2). When abatacept was compared with csDMARDs, there was no significant difference in risk of all malignancies excluding NMSC (IRR 0.97; 95% CI 0.90–1.06) (Supplementary Fig. S6, available at *Rheumatology* online).

### Sensitivity analyses

No significant differences in malignancy risk between abatacept and placebo were observed when analyses were restricted to participants with RA only [RCT/LTE data (IRR 0.66; 95% CI 0.34–1.29); RCT data only (IRR 0.40; 95% CI 0.06–2.55)] (Supplementary Fig. S7, available at *Rheumatology* online). The same observation was found when trial participants at risk of RA were included together with RA and PsA diagnoses (RCT/LTE data: IRR 0.58; 95% CI 0.31–1.08) (Supplementary Fig. S8, available at *Rheumatology* online). Applying a treatment arm continuity correction to the pairwise analysis of eligible combined RCT/LTE data yielded similar results (IRR 0.59; CI 0.29–1.07) (Supplementary Fig. S9, available at *Rheumatology* online). Excluding RCTs deemed to be at a high risk of bias did not change the overall outcome when analysing RCT data alone (IRR 0.29; CI 0.04–2.04); however, removing studies with LTE data that had a high risk of bias resulted in a signal favouring abatacept (IRR 0.38; CI 0.16–0.88) (Supplementary Fig. S10, available at *Rheumatology* online). Estimates were unchanged in the RCT/LTE and observational studies when leave-one-out meta-analyses (Supplementary Figs S11–S13, available at *Rheumatology* online) and influential sensitivity analyses (Supplementary Figs S14–S16, available at *Rheumatology* online) were conducted. Funnel plots were not suggestive of significant publication bias (Supplementary Figs S17–S19, available at *Rheumatology* online); confirmed with Egger’s test of funnel asymmetry (intercept 0.451, 95% CI −0.12–1.02, t = 1.54, *P* =0.15 for RCT/LTE data; intercept 0.29, 95% CI −0.74–1.32, t = 0.55, *P* =0.60 for b/tsDMARDs in the observational data; intercept 0.01, 95% CI −1–1.01, t = 0.02, *P* =0.99 for csDMARDs in the observational data). An additional 32 events in the abatacept arms (number of arms = 14, number of events prior to addition = 88) would have been required for the combined RCT/LTE data to demonstrate that abatacept was significantly associated with harm (IRR 1.81; 95% CI 1.81–3.23).

## Discussion

In a meta-analysis of combined RCT and LTE data, we observed no difference in the risk of all malignancies excluding NMSC between abatacept and placebo or between abatacept and TNFi. However, when meta-analysing observational data, abatacept exposure was associated with an increased risk of malignancy when compared with other b/tsDMARDs, but not compared with csDMARDs.

A previous assessment of malignancy risk with abatacept use in the Truven MarketScan and US Medicare claims databases identified a small increase in overall malignancy risk (*n* = 4328; adjusted hazard ratio 1.17; 95% CI 1.06–1.30), when compared with other b/tsDMARDs [20]. Within this study, NMSC was the only cancer group that demonstrated a significant difference between these treatments (adjusted hazard ratio 1.20; 95% CI 1.03, 1.39). A subsequent comprehensive analysis of the risk of NMSC with abatacept demonstrated a small, but non-significant, safety signal for NMSC risk when abatacept was compared with other b/tsDMARDs using observational data (pooled RR 1.11 0.98–1.26) [21]. Additionally, in this study, when compared with csDMARDs, abatacept was associated with an increased risk of NMSC (pooled RR 1.84, 95% CI 1.00–3.37) [21].

In contrast to NMSC, previous studies have not identified a consistent safety signal for the risk of malignancies excluding NMSC with abatacept [22]. In our study, we observed an increased risk of malignancy when comparing exposure to abatacept with other b/tsDMARDs in observational data. The differences between our findings and previous studies could be explained by the inclusion of a French national claims database study, which had the largest number of patient years of exposure across all drug groups, compared with the evidence that preceded it [11]. This study showed that abatacept use was associated with an increased risk of all cancers excluding NMSC (SIR 1.27; 95% CI 1.16–1.39) and accounted for 32.4% of the overall study weight in our meta-analysis [11].

The contrasting findings between meta-analyses of observational data and trials data in our study demonstrate the importance of real-world data in drug safety evaluations for rare events such as cancer. Tumours have long latency periods. Therefore, the malignancy rates are likely to be lower in the early stages of a clinical trial and increase with the duration of the study. Additionally, study arms with shorter durations of follow-up may be favoured, which could bias towards detecting an observed difference when none exist. This is likely to be compounded by the exclusion of participants displaying symptoms suggestive of malignancy during the screening phase of a clinical trial. In-keeping with this, the RCTs and LTE studies assessed in our study identified no malignancy risk associated with abatacept exposure; however, when analysing observational data with far higher total person-years of exposure, a safety signal was apparent. One potential explanation could be that clinical trials were not powered for the detection of cancers (and other rare events), whereas well-constructed observational studies are better suited to study such signals because of their large sample size and longer duration of follow-up. Importantly, the observational cohorts in our analysis reported standardized incidence ratios but did not adjust for differences between treatment groups. In randomized controlled trials, confounding variables can be assumed to be distributed evenly between treatment groups. In observational data, this assumption may not hold true. In real-world scenarios, abatacept is rarely used as a first line therapy for RA or PsA [43] and so it is possible that differences in key variables such as age, disease duration, disease activity, prior malignancy history, or smoking status may exist when comparing abatacept with other b/tsDMARDs. Therefore, there is likely to be a different baseline cancer risk between patients initiating abatacept and those prescribed other b/tsDMARDs thereby ‘channelling’ patients with a higher baseline cancer risk towards abatacept therapy. Additionally, consideration of diagnostic suspicion and misclassification bias should be considered when interpreting the results of the real-world data in this meta-analysis. In view of the potential biases associated with unadjusted observational data, we cannot draw inferences of causality based upon these findings alone. The optimal method for performing pharmacovigilance of rare adverse events such as cancer remains undetermined. Methods that can incorporate the statistical power of large observational datasets (so-called ‘big data’), whilst accounting for the common biases inherent to observational research, should be incorporated into future studies in this area.

Tumorigenesis associated with abatacept use could be related to the inhibition of CD28, which reduces T-cell activation and can downregulate T-cell responses through unopposed CTLA-4 activity [9]. As such, CD28-blocking therapeutics could impair tumour immune surveillance, providing a biological rationale for our observations. One must also consider the potential contribution of the underlying immune-mediated inflammatory disease [44], disease activity [45] and risk factors such as smoking [46] in overall malignancy risk. Immune-modulating treatments used to control disease can reduce the risk of certain malignancies, such as lymphoma [16], whilst increasing the risk of other cancers; for example, NMSC with the use of mycophenolate mofetil [47]. Taken together, these data highlight the complex interplay between treatment and disease-related factors when trying to elucidate overall malignancy risk with therapies such as abatacept.

The strengths of this study include: (i) the use of the most recent published data from multiple international clinical trials and observational study designs; (ii) combined RCT and LTE data, maximizing the patient-years of exposure and increasing the power to detect differences in rare event, such as cancer; and (iii) the inclusion of data from TNFi and other b/tsDMARD groups, allowed for comparisons between medications characterized by distinct mechanisms of action.

This study also has several limitations. Firstly, the duration of exposure to placebo was relatively short compared with abatacept. Secondly, interpreting the results of trials and observational studies should be done with caution, due to different eligibility criteria used to select populations. Thirdly, heterogeneity is likely to be present between the observational studies, due to differing data collection techniques, reporting standards, processes for reporting events and local prescribing guidelines affecting the order of drug administration. Fourthly, exposure was estimated according to assigned treatment duration when patient-years of exposure was not reported. This approach has been used previously when treatment group exposures were not published; however, there is potential for selection bias [48, 49]. Fifthly, the relatively small number of events by cancer type and inconsistent reporting between studies limited analyses of cancer subtype risk.

In conclusion, abatacept was not associated with a higher incidence of malignancy when compared with placebo or TNFi in RCT/LTE data, as well as when assessed against csDMARDs in observational cohort studies. However, an increased incidence of malignancy was observed with abatacept, when compared with other b/tsDMARDs, in observational data.

## Supplementary Material

keaf114_Supplementary_Data

## Data Availability

Data are available upon reasonable request. All data used in this study are available online within the provided reference list and can be shared upon reasonable request.
