# Supplementary material for: Abatacept and the risk of malignancy: a meta-analysis across disease indications
Source: Rheumatology (Oxford). 2025 Feb 24;64(6):3280–7. doi: 10.1093/rheumatology/keaf114 (PMC12107056; doi:10.1093/rheumatology/keaf114)
Supplement: keaf114_Supplementary_Data [file keaf114_supplementary_data.zip › keaf114_Supplementary_Data/rhe-24-2612-File004.docx]

Supplementary Table S1 Risk of bias randomised controlled trials with or without long-term extension studies


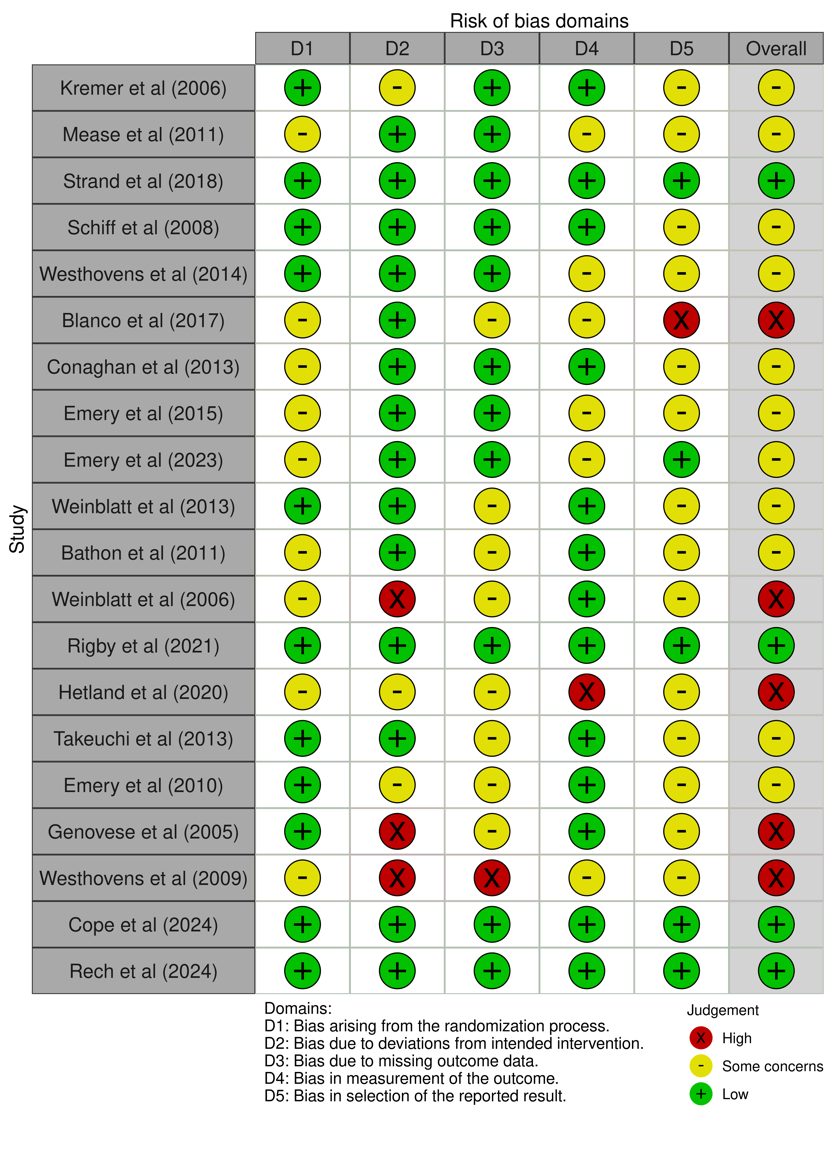


Risk of bias was assessed for each domain using the Cochrane Risk of Bias-2 tool. Green = low risk of bias; yellow = some concerns for bias; red = high risk of bias (24).

Supplementary Table S2. Observational study characteristics – registry data

|  | ARTIS | | | BC | | | FORWARD | | | RABBIT | | | CORRONA | | |
| --- | --- | --- | --- | --- | --- | --- | --- | --- | --- | --- | --- | --- | --- | --- | --- |
| Drug | Abatacept | cs | b/ts | Abatacept | cs | b/ts | Abatacept | cs | b/ts | Abatacept | cs | b/ts | Abatacept | cs | b/ts |
| Country | Sweden | | | Canada | | | USA | | | Germany | | | USA | | |
| Registry started | 1999 | | | 1996 | | | 1998 | | | 2001 | | | 2002 | | |
| Time period (enrolment) | 2007-2016 | | | 1996-2014 | | | 2005-2015 | | | 2007-2017 | | | 2001-2010 | | |
| Mean (SD) follow-up, years | 3.5 (2.4) | 6.2 (3.4) | 4.7 (2.8) | 3.0 (2.1) | 5.9 (4.2) | N/R | 3.2 (2.4) | 2.9 (2.3) | 3.1 (2.5) | 3.7 (2.8) | 3.1 (2.4) | 3.8 (2.6) | 9 | | |
| Number of participants | 2434 | 67762 | 22439 | 637 | 1274 | 4456 | 1496 | 1520 | 3490 | 615 | 3199 | 6810 | 408 | 2470 | 3928 |
| Mean age, years | 59 | 63 | 57 | 58 | 55 | 55 | 62 | 63 | 61 | 58 | 59 | 57 |  |  |  |
| Female participants, % | 80 | 71 | 76 | 84 | 85 | 73 | 86 | 81 | 84 | 75 | 74 | 75 | 84 | 75 | 78 |
| Disease | RA | RA | RA | RA | RA | RA | RA | RA | RA | RA | RA | RA | RA | RA | RA |
| Mean disease duration, years | 14 | N/R | 12 | 10 | 5.2 | 7 | 17 | 15 | 17 | 12 | 6 | 11 | 13 | 9 | 11 |
| Cumulative patient-years of exposure | 6866 | 339977 | 86583 | 1488 | 6246 | 22937 | 2908 | 2808 | 5426 | 4657 | 7064 | 28223 | 589 | 4766 | 10540 |
| Reference | (54) | | | (54) | | | (54) | | | (54) | | | (55) | | |

Supplementary Table S3 Observational studies baseline characteristics – claims databases data

|  |  | MarketScan |  |  | | PharMetrics |  |  | | Optum |  |  | SNDS |  | |
| --- | --- | --- | --- | --- | --- | --- | --- | --- | --- | --- | --- | --- | --- | --- | --- |
|  | Abatacept | cs | b/ts | Abatacept | | cs | b/ts | Abatacept | | cs | b/ts | Abatacept | cs | b/ts | |
| Country |  | | | |  | | | |  | | |  | | |  |
| Mean (SD) follow-up, years | 2.0 | - | 2.2 | 2.4 | | - | 2.7 | 2.3 | | - | 2.4 | 8.7 | | | |
| Number of participants | 17517 | - | 32277 | 12120 | | - | 21145 | 3354 | | - | 5604 | 14559 | 236265 | 94380 | |
| Mean age, years | 55 | - | 54 | 53 | | - | 52 | 51 | | - | 51 |  |  |  | |
| Female participants, % | 82 | - | 83 | 80 | | - | 81 | 82 | | - | 82 | 80 | 74 | 74 | |
| Disease | RA | - | RA | RA | | - | RA | RA | | - | RA | RA | RA | RA | |
| Cumulative patient-years of exposure | 25486 | - | 80929 | 20149 | | - | 71360 | 5914 | | - | 21879 | 36633 | 855321 | 389491 | |
| Reference | (56) | | | (56) | | | | (56) | | | | (16) | | | |

Supplementary Table S4 Observational studies baseline characteristics – cohort study data

| Study | Diep et al (2022) | | Muraoka et al (2021) | |
| --- | --- | --- | --- | --- |
| Drug | Abatacept | b/tsDMARD | Abatacept | csDMARD |
| Country | France | | Japan | |
| Time period | 2005-2018 | | 2014-2018 | |
| Mean (SD) follow-up, years |  |  |  |  |
| Number of participants | 72 | 133 | 114 | 88 |
| Mean age, years | 58.0 | 54.7 | 63.6 | 126.5 |
| Female participants, % | 75 | 81 | 80 | 86 |
| Mean disease duration, years | 13.1 | 9.5 | 8.3 | 7.3 |
| Cumulative patient-years of exposure | 66 | 160 | 53 | 41 |
| Reference | (57) | | (58) | |

Supplementary Table S5 Risk of bias observational studies

| Study title | Author | Selection | Comparability | Outcome | Overall |
| --- | --- | --- | --- | --- | --- |
| Study | Author | Selection | Comparability | Outcome | Overall |
| Comparison of Rheumatoid Arthritis Patients’ 2-Year Infliximab, Abatacept, and Tocilizumab Persistence Rates | Diep et al (2022) | 3 stars | 0 stars | 3 stars | 6 stars |
| Abatacept is Efficacious in the Treatment of Older Patients with csDMARD-Refractory Rheumatoid Arthritis: A Prospective, Multicenter, Observational Study | Muraoka et al (2021) | 4 stars | 1 star | 2 stars | 6 stars |
| Risk of invasive melanoma in patients with rheumatoid arthritis treated with biologics: Results from a collaborative project of 11 European biologic registers | Amari et al (2011) | 4 stars | 2 stars | 3 stars | 9 stars |
| Comparative cancer risk associated with methotrexate, other non-biologic and biologic disease-modifying anti-rheumatic drugs | Solomon et al (2014) | 4 stars | 1 star | 3 stars | 8 stars |
| Malignancy outcomes in patients with rheumatoid arthritis treated with abatacept and other disease-modifying antirheumatic drugs: Results from a 10-year international post-marketing study | Simon et al (2024) | 3 stars | 2 stars | 3 stars | 8 stars |
| Comparative risk of malignancies and infections in patients with rheumatoid arthritis initiating abatacept versus other biologics: a multi-database real-world study | Simon et al (2019) | 3 stars | 2 stars | 3 stars | 8 stars |

Risk of bias was assessed for each domain using the Newcastle-Ottowa scale. Studies are rated from 0-9, rating 0-2 (poor quality), 3-5 (fair quality), 6-9 (good/high quality) (25).

Supplementary Table S6. SUCRA RCT

Surface under the cumulative ranking curve (SUCRA) method to rank malignancy risk between treatments from network meta-analyses. Each treatment was ranked based upon the estimated probability (%) of it causing the least number of malignancies under the cumulative ranking curve. This was derived from network meta-analyses of RCT/LTE data. Higher SUCRA values indicate a greater likelihood of a given treatment causing the least number of malignancies, such that when the SUCRA value is 1, the treatment is certain to be the best, and when it is 0, it is certain to be the worst.

| Drug | P-score | Rank |
| --- | --- | --- |
| Abatacept | 0.64 | 1 |
| TNFi | 0.59 | 2 |
| Placebo | 0.27 | 3 |

Supplementary Table S7. SUCRA RCT/LTE

Surface under the cumulative ranking curve (SUCRA) method to rank malignancy risk between treatments from network meta-analyses. Each treatment was ranked based upon the estimated probability (%) of it causing the least number of malignancies under the cumulative ranking curve. This was derived from network meta-analyses of RCT/LTE data. Higher SUCRA values indicate a greater likelihood of a given treatment causing the least number of malignancies, such that when the SUCRA value is 1, the treatment is certain to be the best, and when it is 0, it is certain to be the worst.

| Drug | P-score | Rank |
| --- | --- | --- |
| Abatacept | 0.86 | 1 |
| TNFi | 0.43 | 2 |
| Placebo | 0.21 | 3 |
