# Supplementary material for: Abatacept and the risk of malignancy: a meta-analysis across disease indications
Source: Rheumatology (Oxford). 2025 Feb 24;64(6):3280–7. doi: 10.1093/rheumatology/keaf114 (PMC12107056; doi:10.1093/rheumatology/keaf114)
Supplement: keaf114_Supplementary_Data [file keaf114_supplementary_data.zip › keaf114_Supplementary_Data/rhe-24-2612-File005.docx]

Eligibility

Records identified through database searches

(n = 1458)

Records after duplicates removed

(n = 1272)

Records screened

(n = 1272)

Records excluded (n = 1217), due to:

- Ineligible study type
- No abatacept data
- No safety data
- Duplicate study

Full-text articles assessed for eligibility

(n = 55)

Full text articles excluded (n = 38), due to:

- No malignancy data
- More contemporary safety data available
- Insufficient follow-up
- Study population overlap

Studies included in meta-analysis

(n = 17)

Screening

Identification

**Supplementary Figure S1.**

Flowchart of studies identified in the systematic literature search for RCT/LTE data. RCT: randomised controlled trial; LTE: long-term extension

Eligibility

Records identified through database searches

(n = 1092)

Records after duplicates removed

(n = 1030)

Records screened

(n = 1030)

Records excluded (n = 1016), due to:

- Ineligible study type
- No abatacept data
- No safety data
- Duplicate study

Full-text articles assessed for eligibility

(n = 14)

Full text articles excluded (n = 8), due to:

- No malignancy data
- More contemporary safety data available
- Study population overlap

Studies included in meta-analysis

(n = 6)

Screening

Identification

**Supplementary Figure S2.**

Flowchart of studies identified in the systematic literature search for observational data.

**Supplementary Figure S3.**

Pairwise meta-analysis of the risk of all malignancies excluding non-melanomatous skin cancers between abatacept and placebo groups of eligible RCT (top panel) and combined RCT and LTE studies (bottom panel); expressed as incidence rate ratios with 95% CIs and depicted graphically as a forest plot. Exposure is reported in person-years. The relative weighting of each study from a random-effects model is shown. A treatment arm continuity correction was applied to all studies. Heterogeneity between studies was assessed using I^2^ statistics. CI, confidence interval; LTE, long-term extension; RCT, randomised controlled trial.

**Supplementary Figure S4.**

Network plot, depicting the number of studies for each treatment (node size) and number of treatment comparisons (edge thickness) in eligible RCT studies. Network meta-analysis estimates of the risk of all malignancies excluding non-melanomatous skin cancers between study treatments expressed as incidence rate ratios with 95% CIs and depicted graphically as a forest plot. CI, confidence interval; LTE, long-term extension; RCT, randomised clinical trial; TNFi, tumour necrosis factor-α inhibitor.

**Supplementary Figure S5.**

Pairwise meta-analysis of the risk of all malignancies excluding non-melanomatous skin cancers between abatacept and TNFi groups of eligible RCT (top panel) and combined RCT/LTE (bottom panel) studies; expressed as incidence rate ratios with 95% CIs and depicted graphically as a forest plot. Exposure is reported in person-years. The relative weighting of each study from a random-effects model is shown. A fixed continuity correction of 0.1 was applied to all studies with zero events. Heterogeneity between studies was assessed using I^2^ statistics. CI, confidence interval; LTE, long-term extension; RCT, randomised controlled trial; TNFi, tumour necrosis factor-α inhibitor.

**Supplementary Figure S6.**

Pairwise meta-analysis of the risk of all malignancies excluding non-melanomatous skin cancers between abatacept and conventional synthetic disease modifying anti-rheumatic drug groups of eligible observational cohort studies; expressed as incidence rate ratios with 95% CIs and depicted graphically as a forest plot. Exposure is reported in person-years. The relative weighting of each study from a random-effects model is shown. A fixed continuity correction of 0.1 was applied to all studies with zero events. Heterogeneity between studies was assessed using I^2^ statistics. CI, confidence interval; csDMARD, conventional synthetic disease modifying anti-rheumatic drugs.

**Supplementary Figure S7.**

Sensitivity network meta-analysis of the risk of all malignancies excluding non-melanomatous skin cancers between abatacept and placebo groups of eligible RCT (left panel) and combined RCT/LTE studies (right panel) only with RA populations; expressed as incidence rate ratios with 95% CIs and depicted graphically as a forest plot. Exposure is reported in person-years. The relative weighting of each study from a random-effects model is shown. A fixed continuity correction of 0.1 was applied to all studies with zero events. Heterogeneity between studies was assessed using I^2^ statistics. CI, confidence interval; LTE, long-term extension; RCT, randomised controlled trial.

**Supplementary Figure S8.**

Sensitivity network meta-analysis of the risk of all malignancies excluding non-melanomatous skin cancers between abatacept and placebo groups of eligible combined RCT/LTE studies of all treatment indications including trials with pre-RA populations; expressed as incidence rate ratios with 95% CIs and depicted graphically as a forest plot. Exposure is reported in person-years. The relative weighting of each study from a random-effects model is shown. A fixed continuity correction of 0.1 was applied to all studies with zero events. Heterogeneity between studies was assessed using I^2^ statistics. CI, confidence interval; LTE, long-term extension; RCT, randomised controlled trial.

**
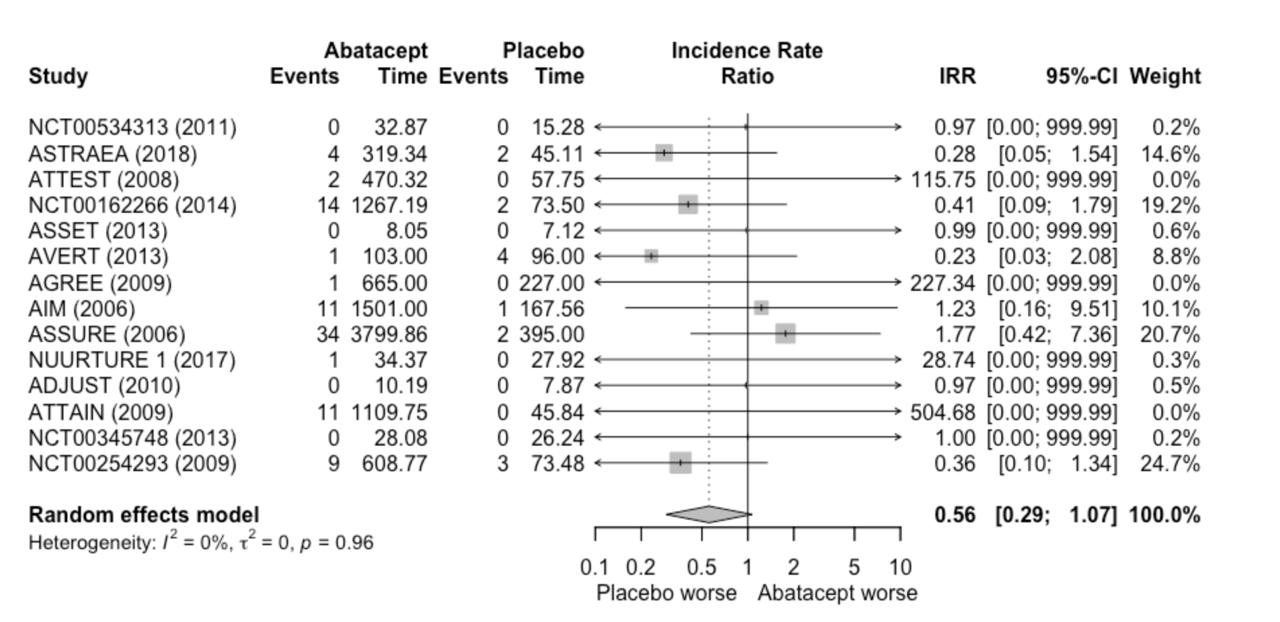
Supplementary Figure S9.**

Sensitivity pairwise meta-analysis of the risk of all malignancies excluding non-melanomatous skin cancers between abatacept and placebo groups of eligible combined RCT/LTE studies; expressed as incidence rate ratios with 95% CIs and depicted graphically as a forest plot. Exposure is reported in person-years. The relative weighting of each study from a random-effects model is shown. A treatment arm continuity correction was applied to all studies with zero events. Heterogeneity between studies was assessed using I^2^ statistics. CI, confidence interval; LTE, long-term extension; RCT, randomised controlled trial.

**Supplementary Figure S10.**

Sensitivity network meta-analysis of the risk of all malignancies excluding non-melanomatous skin cancers between abatacept and placebo groups excluding studies with a high risk of bias from RCT (top panel) and RCT/LTE data (bottom panel); expressed as incidence rate ratios with 95% CIs and depicted graphically as a forest plot. Exposure is reported in person-years. The relative weighting of each study from a random-effects model is shown. A fixed continuity correction of 0.1 was applied to all studies with zero events. Heterogeneity between studies was assessed using I^2^ statistics. CI, confidence interval; LTE, long-term extension; RCT, randomised controlled trial.

**Supplementary Figure S11.**

Sensitivity analysis demonstrating the influence of individual studies on pairwise meta-analysis results for all malignancy events, comparing abatacept with placebo groups of eligible combined RCT and LTE studies. The effect estimates (incidence rate ratio and 95% CIs) provided each row correspond to the pairwise meta-analysis results when excluding that study. A fixed continuity correction of 0.1 was used for studies with zero events. CIs: confidence intervals; RCT: randomised controlled trial; LTE: long-term extension.

**Supplementary Figure S12.**

Sensitivity analysis demonstrating the influence of individual studies on pairwise meta-analysis results for all malignancy events, comparing abatacept with b/tsDMARD groups of eligible observational studies. The effect estimates (incidence rate ratio and 95% CIs) provided each row correspond to the pairwise meta-analysis results when excluding that study. A fixed continuity correction of 0.1 was used for studies with zero events. b/tsDMARD: biologic/targeted synthetic disease modifying anti-rheumatic drug; CIs: confidence intervals.

**Supplementary Figure S13.**

Sensitivity analysis demonstrating the influence of individual studies on pairwise meta-analysis results for all malignancy events, comparing abatacept with csDMARD groups of eligible observational studies. The effect estimates (incidence rate ratio and 95% CIs) provided each row correspond to the pairwise meta-analysis results when excluding that study. A fixed continuity correction of 0.1 was used for studies with zero events. csDMARD: biologic/targeted synthetic disease modifying anti-rheumatic drug; CIs: confidence intervals.

**Supplementary Figure S14.**

Sensitivity analysis demonstrating the influence of individual studies on pairwise meta-analysis results for all malignancy events, comparing abatacept with placebo groups of eligible combined RCT and LTE studies. Baujat plot (top panel) to identify studies contributing to heterogeneity. Studies in the top right quadrant have a greater influence on the overall result and contribute most to study heterogeneity. Influence diagnostics plots (bottom panel) displaying externally standardised results, DFFITS values, Cook’s distance, covariance ratios, leave-one-out τ^2^ and Cochran’s Q values, hat value and study weight.

**Supplementary Figure S15.**

Sensitivity analysis demonstrating the influence of individual studies on pairwise meta-analysis results for all malignancy events, comparing abatacept with b/tsDMARD groups of eligible observational studies. Baujat plot (top panel) to identify studies contributing to heterogeneity. Studies in the top right quadrant have a greater influence on the overall result and contribute most to study heterogeneity. Influence diagnostics plots (bottom panel) displaying externally standardised results, DFFITS values, Cook’s distance, covariance ratios, leave-one-out τ^2^ and Cochran’s Q values, hat value and study weight. b/tsDMARD: biologic/targeted synthetic disease modifying anti-rheumatic drug.

**Supplementary Figure S16.**

Sensitivity analysis demonstrating the influence of individual studies on pairwise meta-analysis results for all malignancy events, comparing abatacept with csDMARD groups of eligible observational studies. Baujat plot (top panel) to identify studies contributing to heterogeneity. Studies in the top right quadrant have a greater influence on the overall result and contribute most to study heterogeneity. Influence diagnostics plots (bottom panel) displaying externally standardised results, DFFITS values, Cook’s distance, covariance ratios, leave-one-out τ^2^ and Cochran’s Q values, hat value and study weight. csDMARD: conventional synthetic disease modifying anti-rheumatic drug.

**Supplementary Figure S17.**

Funnel plots, assessing potential publication bias in pairwise meta-analysis, comparing abatacept and placebo groups of eligible combined RCT and LTE data. RCT: randomised controlled trial; LTE: long-term extension.

**Supplementary Figure S18.**

Funnel plots, assessing potential publication bias in pairwise meta-analysis, comparing abatacept and b/tsDMARD groups of eligible observational data. b/tsDMARD: biologic/targeted synthetic disease-modifying anti-rheumatic drug.

**Supplementary Figure S19.**

Funnel plots, assessing potential publication bias in pairwise meta-analysis, comparing abatacept and csDMARD groups of eligible observational data. csDMARD: conventional synthetic disease-modifying anti-rheumatic drug.
